# Estimates for Genetic Variance Components in Reciprocal Recurrent Selection in Populations Derived from Maize Single-Cross Hybrids

**DOI:** 10.1155/2014/540152

**Published:** 2014-06-09

**Authors:** Matheus Costa dos Reis, José Maria Villela Pádua, Guilherme Barbosa Abreu, Fernando Lisboa Guedes, Rodrigo Vieira Balbi, João Cândido de Souza

**Affiliations:** ^1^DuPont do Brasil S.A., Divisão Pioneer Sementes, 73310-970 Brasília, DF, Brazil; ^2^Departamento de Biologia (DBI), Universidade Federal de Lavras (UFLA), Lavras, MG, Brazil; ^3^Embrapa Cocais, São Luis, MA, Brazil; ^4^Embrapa Semi-Árido, Sobradinho, CE, Brazil; ^5^Departamento de Agricultura (DAG), Universidade Federal de Lavras (UFLA), Lavras, MG, Brazil

## Abstract

This study was carried out to obtain the estimates of genetic variance and covariance components related to intra- and interpopulation in the original populations (C0) and in the third cycle (C3) of reciprocal recurrent selection (RRS) which allows breeders to define the best breeding strategy. For that purpose, the half-sib progenies of intrapopulation (P_11_ and P_22_) and interpopulation (P_12_ and P_21_) from populations 1 and 2 derived from single-cross hybrids in the 0 and 3 cycles of the reciprocal recurrent selection program were used. The intra- and interpopulation progenies were evaluated in a 10 × 10 triple lattice design in two separate locations. The data for unhusked ear weight (ear weight without husk) and plant height were collected. All genetic variance and covariance components were estimated from the expected mean squares. The breakdown of additive variance into intrapopulation and interpopulation additive deviations (*σ*_*τ*_^2^) and the covariance between these and their intrapopulation additive effects (Cov_*Aτ*_) found predominance of the dominance effect for unhusked ear weight. Plant height for these components shows that the intrapopulation additive effect explains most of the variation. Estimates for intrapopulation and interpopulation additive genetic variances confirm that populations derived from single-cross hybrids have potential for recurrent selection programs.

## 1. Introduction

Reciprocal recurrent selection (RRS) was originally proposed by Comstock et al. [1] to improve the hybrid between two populations to exploit additive and nonadditive effects. This method also allows the improved populations to be the source of inbred lines, producing superior hybrids when compared to hybrids obtained from the original populations [2, 3].

Since the RRS has been proposed, numerous studies in the literature have been designed to clarify particular aspects of this selection method, such as a suggestion for changes in the original method [4–6], comparisons between methods [7, 8], and evidence of the effectiveness of these methods in improving interpopulation crosses [9–13]. In many situations, there were reports that these interpopulation selection methods significantly increased the response in the interpopulation hybrid and in one of the populations, with an unsatisfactory or even negative response in the other population [14].

One of the main points to be attentive to in reciprocal recurrent selection is maintenance of the variability of the population in each breeding cycle. Thus, estimates of genetic variance components are useful for breeding purposes because they allow the use of appropriate strategies to achieve success in breeding programs.

Alternatives to obtaining estimates of these variance components are detailed in some publications [15–17]. Júnior [18] proposed new genetic variance and covariance components, as well as a new method called testcross half-sib selection (THS). This author suggested obtaining estimates for such components from the different recurrent selection methods for the original populations (C0) and the improved populations (cycle* n*) and verifying their magnitude and changes from selection. In the few cases in which these components were estimated, only the original populations (C0) and the interpopulation hybrid between them were involved [19, 20]. Therefore, it is necessary to obtain more estimates in order to make accurate inferences about this method and, more importantly, to obtain estimates of variance from the populations in advanced cycles of selection.

The present study was carried out in order to obtain estimates for genetic variance and covariance components of the original populations (C0) and third cycle (C3) of an RRS program.

## 2. Materials and Methods

### 2.1. Maize Populations

Reciprocal recurrent selection began with two populations (1 and 2), each of which was derived from one commercial single-cross hybrid that in prior diallel test was demonstrated to have a great potential to generate good hybrids (combine well) [21]. Each population was obtained by random intercrossing of 3000 F1 hybrid plants (to fill full the area) in separate fields. The two S0 populations (P1 and P2) were assumed to be in Hardy-Weinberg equilibrium [16].

The method used in the SRR program is similar to that proposed by Júnior [6]. In the first selection cycle (cycle 0—C0), 2000 plants were sown by population, in which the prolific plants of both populations had their down ears self-pollinated and upper ears were crossed with plants of the reciprocal population, which generated S1 progeny and progeny of interpopulation sibs in each genotype (plant). The seeds of S1 progenies were reserved to compose the unit recombination, and full-sib progenies were used for the evaluation of progenies. 121 interpopulational progenies were evaluated in a simple lattice 11 × 11. We selected 10% of the progeny, and the average productivity (weight of husked ears) was mainly considered. To facilitate the completion a faster cycle, it was necessary to sow the recombination field in winter.

Progenies of the second cycle (C1—cycle 1) were obtained. Differently of the previous cycle (C0) in Cicle 1 (C1) were obtained in prolific plants, in intrapopulation half-sib progenies in the down ears, and in the upper ears interpopulation half sib, using a mixture of pollen of the population and the reciprocal population, respectively. One hundred plants of each population, that presented good aspect and with good grain filling in both ears, were sampled to obtain the intra- and interpopulation progenies. The 100 progenies of each population of C1 were evaluated in experiments conducted in the 10 × 10 lattice design with three replications, following the same procedures performed in the previous harvest. The 15% progenies with more productivity of husked ears were selected.

Using the seeds of progeny intrapopulation half-sib progenies related to interpopulation selected in each population, the third round selection (Cycle 2—C2) began. The same procedure utilized in (C) was performed, which generated again S1 progenies and full-sib progenies of interpopulation. After the field evaluation the 15% best progenies were selected which generated the third cicle (C3).

The use of alternate progenies between full-sib and half-sib progenies in this SRR program was to evaluate the efficiency between the methods and to estimate the variances and covariances so that we could better study the RSS program. More details of the selection procedures can be seen in Reis et al. [22].

To evaluate the efficiency of this RSS program, progenies from two cycles (C0 and C3) were employed. One hundred intra-half-sib hybrids from population 1 from each cycle were evaluated, the same number from population 2 (P_11_ and P_22_), and 100 interpopulation half-sib hybrid progenies with population 1 (P_12_) and population 2 (P_21_) as female. To obtain these progenies, 2000 seeds from each population were sown. For this, down ears were pollinated with bulk pollen from the reciprocal population (interpopulation half sib) and the up ears were pollinated with bulk pollen from the same population (intrapopulation half sib). Thus, eight types of progenies were evaluated. The present study involved the evaluation of half-sib progenies in cycles of average length of 18 months.

### 2.2. Estimation of Variance and Covariance Components

In the 2007/2008 crop season, the experiments were set up in the experimental fields of the Department of Biology (DBI) in Lavras, MG, Brazil (21°14′S, 45°00′W; altitude of 918 m), and at the UFLA Experimental Farm located in Ijaci, MG (21°10′S, 44°55′W; altitude of 951 m). In each location, the progenies were evaluated in different experiments, side by side. In each case, a 10 × 10 triple lattice experimental design was used. The plots consisted of two 3-meter rows at a spacing of 0.80 m and the distance between plants was 0.23 m, for a total of 55000 plant·ha^−1^. The traits evaluated were unhusked (without husk) ear weight (EW—g/plant) and plant height (PH—cm/plant). Ear weight was determined for grain moisture of 14%. Plant height was obtained from the average of five competitive plants.

Data were subjected to analyses of variance for individual and combined locations based on adjusted mean values for each type of progeny. The mean value and the site effect were considered as fixed and the other effects were considered as random. Subsequently, analyses of covariance between intra- and interpopulation progenies for each C0 and C3 population were performed. All analytical procedures were carried out using the SAS computer application [23]. Variances and covariances were calculated from the expected values of the intrapopulational (*Q*_*A*_) and interpopulational (*Q*_*E*_) mean squares and the mean products (*P*_*AE*_) of the C0 and C3 cycles.

Mean estimates for genetic variance and covariance among progeny in individual and combined analysis (*σ*_*P*_^2^ and cov⁡_*P*_) were obtained from the mean squares (*Q*_*p*_) and mean products (*P*_*p*_) of the progenies and from the mean squares (*Q*_pxa_) and mean products (*P*_pxa_) of the progeny *x* location interactions, according to the following expressions: *σ*_*P*_^2^ = (*Q*_*p*_ − *Q*_pxa_)/*rl* and cov⁡_*p*_ = (*P*_*p*_ − *P*_pxa_)/*rl*, where *r* and *l* are the numbers of repetitions and locations, respectively. From *σ*_*P*_^2^, the estimates of intrapopulation additive genetic variance of populations 1 and 2 (*σ*_*A*11_^2^ and *σ*_*A*22_^2^) and interpopulation additive variance (*σ*_*A*12_^2^ and *σ*_*A*21_^2^) were estimated, according to Júnior [18]. Interpopulation additive variance means that the genetic variances and covariances represent the mean expected value of a population or of a population cross and are expressed as a function of *a* and *d* genotypic values [6].

Júnior [18] demonstrates that genetic variance expressed as the intersection of the two populations 1 and 2 (*σ*_*P*21_^2^) is provided by the expression

(1) $$\begin{array}{c} \sigma_{G12}^{2}=\frac{1}{2}\left(\sigma_{A12}^{2}+\sigma_{A21}^{2}\right)+\sigma_{D12}^{2}, \end{array}$$

where *σ*_*A*12_^2^ and *σ*_*A*21_^2^ are the interpopulation additive genetic variance with populations 1 and 2 as female parents, respectively, and *σ*_*D*12_^2^ is the dominant interpopulation genetic variance.

Interpopulation components can also be expressed as a function of the magnitude of the allelic frequencies of the two populations involved and the type of allelic interaction. Considering only one locus with two alleles, we have

(2) σA122=2p(1−p)[a  +(1−2r)d]2;σA122=2r(1−r)[a+(1−2p)d]2,σD122=4pqrsd2.

In these expressions, *p* and *r* refer to the frequencies of favorable alleles in populations 1 and 2, respectively; *q* and *s* refer to the frequency of unfavorable alleles under the same conditions; *a* is the contribution of homozygous loci; and *d* is the genotypic value of the heterozygote.

The interpopulation additive variances were partitioned into the following components: intrapopulation additive variance, variances of the deviations of the intra- by interpopulation additive effects (*σ*_*τ*12_^2^ and *σ*_*τ*21_^2^), and covariance of these deviations with the additive effects ${\text{C}\hat{\text{o}}\text{v}}_{A1\tau 12}$ and ${\text{C}\hat{\text{o}}\text{v}}_{A2\tau 21}$, according to Júnior [18]. These estimates were obtained using the procedure of iterative weighted least squares *β* = (*X*′*V* ^−1^ *X*)^−1^ *X*′*V* ^−1^ *Y* and the standard error came from the square root of diagonal matrix (*X*′*V* ^−1^ *X*)^−1^, similar to the procedure presented by Arias and Júnior [19].

Estimates of heterosis in cycles C0 and C3 were obtained from the mean yield values of the parental populations. The realized direct and indirect responses (defined as the performances of the interpopulational hybrids and of the populations per se, resp.) to RRS were obtained from the differences between the mean productivity values of the C3 and C0 interpopulational hybrids and the populations per se, respectively. The differences were divided by the mean yields in C0 and the values were expressed as percentages.

## 3. Results and Discussion

In combined analysis of variance for unhusked ear weight (EW—g/plant) in each type of progeny (intra- and interpopulational) of populations 1 and 2, significant differences were detected (*P* < 0.05) among the progenies (P) in almost all situations (Table 1). Considering the plant height trait (PH cm/plant), the results were even better. There was no significant difference (*P* < 0.05) only for MS_22 _in population 2 (C0) (Table 2). These results confirm the presence of significant variance among the progenies of both populations and indicate that the parent that gave rise to them must have a large number of heterozygous loci. The precision of the experiments measured by the coefficient of variation (CV) indicates that all evaluations have good precision. This is the most important requisite for good interpretation of the results.

Estimates for intrapopulation additive genetic variance (${\hat{\sigma }}_{A}^{2}$) in cycle zero were higher for population 2, except for plant height, which was negative (Table 3). The estimate for intrapopulation additive variance of population 2 (${\hat{\sigma }}_{A22}^{2}$) for unhusked ear weight is above the average of 58 estimates (${\hat{\sigma }}_{A}^{2}=309.0$) reported by Vencovsky et al. [24], while the estimate obtained for population 1 was slightly lower. Using the same population 1, Raposo and Ramalho [20] observed a much lower estimate (${\hat{\sigma }}_{A}^{2}=49.44$) compared to this study. The ${\hat{\sigma }}_{A11}^{2}$ for plant height was below the average obtained from 16 populations (${\hat{\sigma }}_{A}^{2}=321.0$) reported by Filho [25] and similar to the estimate obtained from ${\hat{\sigma }}_{A}^{2}=185.74$ by Arias and Júnior [19]. These results indicate that the populations derived from single-cross hybrids show enough additive genetic variance to allow success in intrapopulation breeding programs.

Estimates for interpopulation additive genetic variances from cycle zero, as well as intrapopulation additive genetic variances, were higher for population 2 (Table 3). The estimate for interpopulation additive variance of ear weight from population 1 (${\hat{\sigma }}_{A12}^{2}$) was low, with a high level of associated error. Despite this, the average estimate for interpopulation additive variance of populations 1 and 2 for ear weight $\left[{\hat{\sigma }}_{A(12)}^{2}=\left(1/2\right)\left({\hat{\sigma }}_{A12}^{2}+{\hat{\sigma }}_{A21}^{2}\right)=345.16\right]$ was higher than the average of 58 estimates (mean of 203.9) described by Vencovsky et al. [24]. For plant height, the average of interpopulation additive genetic variances (${\hat{\sigma }}_{A(12)}^{2}=188.5$) was similar to the average of 193.5 obtained in other situations [19, 25]. These results confirm that the populations involved have great potential for a reciprocal recurrent selection program.

In general, ${\hat{\sigma }}_{\tau }^{2}$ presented higher values for population 2 and low level of error associated with both C0 populations (Table 3). The only estimate that showed a high level of associated error was ${\hat{\sigma }}_{\tau 12}^{2}$ for EW and even considering the lowest absolute value (60.5) this estimate is in, it is still higher than the estimates obtained (${\hat{\sigma }}_{\tau 12}^{2}=37.6$ and ${\hat{\sigma }}_{\tau 21}^{2}=47.0$) by Arias and Júnior [19]. In other estimates found in the literature, Raposo and Ramalho [20], also working with populations from single-cross hybrids, reported ${\hat{\sigma }}_{\tau 12}^{2}=-121.85$ and −199.37 and ${\hat{\sigma }}_{\tau 21}^{2}=148.08$ and 110.11 for ear weight and plant height in populations 1 and 2, respectively. Arias and Júnior [19] found estimates lower than those reported in this study for ear weight and plant height. The genetic significance of στ2 can be better understood when its expression is observed as a function of the allele frequencies of the populations involved and the presence of dominance; that is, στ122=8pq(p-r)2d2 and *σ*_*τ*12_^2^ = 8*rs*(*p*−*r*)^2^ *d* ^2^. In these expressions, *p* and *r* refer to the frequency of favorable alleles in populations 1 and 2, respectively, and *q* and *s* to the frequency of unfavorable alleles in the populations. As for *d*, it represents the value of the heterozygous genotype or dominance effect. Therefore, the direct relationship with heterosis is made very clear.

It may therefore be inferred that the populations involved are divergent, with the presence of dominance for the traits in question, which is a condition for the existence of heterosis and also, as mentioned, the potential for a reciprocal recurrent selection program. In fact, heterosis was 12.3% in cycle 0 and 24.9% in cycle 3 (Table 4) showing the potential for RRS in the populations.

Regarding the covariance of additive effects with their intra- by interpopulation deviations in cycle zero, all estimates were negative and in some cases with high levels of error (Table 3). Arias and Júnior [19] reported a similar situation with negative estimates (${\text{C}\hat{\text{o}}\text{v}}_{A1\tau 12}$) of −41.08 and −8.90 and (${\text{C}\hat{\text{o}}\text{v}}_{A1\tau 12}$) of −88.41 and −44.67 for ear weight and plant height, respectively, for the two populations studied. In another report, Raposo and Ramalho [20] obtained negative estimates for ear weight and plant height in both populations involved (${\text{C}\hat{\text{o}}\text{v}}_{A1\tau 12}=-208.08$ and −17.32 and ${\text{C}\hat{\text{o}}\text{v}}_{A2\tau 21}=-7.27$ and −25.48). These covariances are determined by the expressions described by Júnior [18] {Cov_*A*1*τ*12_ = 2*pq*(*p* − *r*)[*a* + (1 − 2*p*)*d*]*d* and Cov_*A*2*τ*21_ = 2*rs*(*r* − *p*)[*a* + (1 − 2*r*)*d*]*d*}, where *a* is the deviation of homozygotes in relation to the midpoint. Therefore, it is expected that, for a location to contribute to these covariances, genetic divergence (*p* ≠ *r*) and the presence of dominance are necessary, and, consequently, the covariance would be positive for the population with a higher average frequency of favorable alleles and negative for the population with a lower average frequency for these alleles. However, there are a large number of loci which one can cancel another. There are some cases in which loci are fixed in both populations (*p* = 1.0 and *r* = 1.0 or *p* = 1.0 and *r* = 0.0) [20]. Therefore, it is difficult to draw conclusions regarding genetic composition of the populations from covariance estimates.

Comparing the estimates for intrapopulation additive genetic variances in cycle zero to the ones in cycle three, a reduction in ear weight in population 2 and an increase in population 1 were observed. However, the estimates for C0 and C3 were within the limits of standard error only in population 1 and thus did not differ from each other. As for PH, σ^A222, which was negative in C0, was significantly higher than the same estimate for population 1 in C3. In a study comparing the additive genetic variance in populations BSSS and BSCB after four RRS cycles, Hallauer [9] reported similar results for EW. However, a compilation after eight RRS cycles in this program showed no changes in genetic variability from selection [15].

The results obtained here, together with those reported in the literature, show that, during an RRS program, intrapopulation genetic variances can vary in magnitude without compromising the genetic variability of populations, thus leading to success of the populations involved in breeding programs.

As for the interpopulation additive variances of C3 (Table 3), it may be observed that the estimate for EW of population 2 was twice as high as C0. Despite the increase in the same estimate for population 1, the level of error was very high, indicating that this may be zero or negative, but for PH there were no changes. From these results, we can infer that there was an increase of genetic variability, especially for EW, the most important trait in maize cropping. Several reports were found in the literature, corroborating the results obtained in this study [26, 27]. Therefore, genetic gains are expected in future cycles with interpopulation selection.

Maintenance of genetic variability during the recurrent selection process is important. In this respect, the results described in the literature and these obtained in this study showed an increase in this variability, especially in the initial cycles. One explanation for this is that, at the recombination stages, gene blocks are broken up and, with each recombination, greater genetic variability is generated in populations of advanced cycles compared to the initial populations.

Analyzing the σ^τ2 obtained for the C3 populations, an increase in their magnitudes for EW was noticed compared to estimates obtained from the C0 populations. There was a decrease in PH, with a more pronounced decrease in the estimate for population 1. The estimates for $\text{C}\hat{\text{o}}{\text{v}}_{A\tau }$ were also negative or with a high level of error, which does not allow inferences to be made in regard to the sign of the estimate. In both populations, $\text{C}\hat{\text{o}}{\text{v}}_{A\tau }$ had much lower magnitudes than the estimates of intrapopulation additive variance, indicating that additive effects explained most of the variation for these traits. Therefore, comparisons between Cov_(*Aτ*)_ of the original populations (C0) and those of C3 are justified only for the ear production trait. It may be noted that the magnitudes in module increased for population 1 and decreased for population 2 after three selection cycles. Unfortunately, no reports of these components (${\hat{\sigma }}_{\tau }^{2}$) in populations of advanced selection cycles were found for comparison.

According to a theoretical study, Júnior [18] showed that an increase in the magnitude of these components with RRS is expected for traits with an average degree of dominance *d*/*a* from 0.75 to 1.25 (e.g., grain yield) and, for characters with *d*/*a* near or below 0.5 (e.g., plant height), the components may be disregarded. Overall, the results obtained in this study are consistent with expectations. Estimates of ${\hat{\sigma }}_{\tau }^{2}$ and $\text{C}\hat{\text{o}}{\text{v}}_{A\tau }$ increased for EW from the selection process, while for AP there was a decrease.

It is possible that the progeny by environment interaction, in this case, has impaired the accuracy of ${\hat{\sigma }}_{\tau }^{2}$ and $\text{C}\hat{\text{o}}{\text{v}}_{A\tau }$ because these estimates are obtained from the mean squares and products of the analyses for intra- and interpopulation progenies and therefore are subject to the adversities that occur in conducting experiments involving large numbers of progenies and types of progenies. The errors associated with the components, in some cases, were high because there is dependency between the estimated parameters (${\hat{\sigma }}_{\tau }^{2}$ and $\text{C}\hat{\text{o}}{\text{v}}_{A\tau }$) and the variables used (mean squares and products). The iterative process, which is an attempt to mitigate this problem, converged at only one iteration since the number of equations used in the model is equal to the number of parameters estimated (components). Including Genotype per environment in this study is impossible because there were more parameters than equations to estimate.

## 4. Conclusions

Estimates for intrapopulation and interpopulation additive genetic variances confirm that populations derived from single-cross hybrids have potential for recurrent selection programs.

There was an increase in the mean values of additive effects for EW in population 2 and a decrease in population 1 after three RRS cycles.

RRS improved the complementarity of the interpopulational hybrid such that heterosis increased from 12.3% to 24.9% after three cycles.

## Figures and Tables

**Table 1:** Combined analysis of variance for unhusked ear weight (EW—g/plant) to evaluate progenies of intra- and interpopulation half sibs from cycle 0 (C0) and cycle 3 (C3).

VF	DF	C0				C3			
		Population 1		Population 2		Population 1		Population 2	
		MS_11_	MS_12_	MS_22_	MS_21_	MS_11_	MS_12_	MS_22_	MS_21_
Environment (E)	1	32545.2^ns^	76526.41*	31947.0^ns^	118584.2*	132336.5*	280837.4*	93620.8^ns^	221664.09*
Progeny (P)	99	1372.62*	1265.74^ns^	1453.06**	1584.65**	1260.23**	1986.26^ns^	1156.75^ns^	2990.09**
E × P	99	996.99**	1224.85**	472.23^ns^	684.19^ns^	730.92**	1725.41^ns^	891.26**	1083.31^ns^
Error	342	622.94	553.30	494.33	556.97	441.26	1382.01	600.17	1062.86

CV (%)		14.99	11.99	12.65	12.18	15.19	19.21	14.45	16.92
Means		166.49	186.16	175.76	183.66	138.10	193.47	169.55	192.66

*F test is significant at 5%.

**F test is significant at 1%.

^ns^ *F* test is nonsignificant.

**Table 2:** Combined analysis of variance for plant height (PH cm/plant) to evaluate progenies of intra- and interpopulation half sibs from cycle 0 (C0) and cycle 3 (C3).

VF	DF	C0				C3			
		Population 1		Population 2		Population 1		Population 2	
		MS_11_	MS_12_	MS_22_	MS_21_	MS_11_	MS_12_	MS_22_	MS_21_
Environment (E)	1	231673.5*	195662.0**	132165.0*	145085.3*	8361.04^ns^	1292.13^ns^	8970.67^ns^	542.94^ns^
Progeny (P)	99	449.97**	474.68**	242.40^ns^	518.69**	353.91**	446.42**	581.38**	563.51**
E × P	99	192.59^ns^	230.10^ns^	247.09^ns^	249.18^ns^	226.26^ns^	245.25^ns^	213.70^ns^	279.21^ns^
Error	342	152.56	212.92	231.85	295.41	251.21	228.99	281.68	253.07

CV (%)		6.27	7.05	7.59	8.32	8.94	7.36	8.64	7.70
Means		196.97	206.79	200.46	206.48	177.35	205.55	194.17	206.58

*F test is significant at 5%.

**F test is significant at 1%.

^ns^ *F* test is nonsignificant.

**Table 3:** Estimates for intrapopulation (${\hat{\sigma }}_{A11}^{2}$ and ${\hat{\sigma }}_{A22}^{2}$) and interpopulation (${\hat{\sigma }}_{A12}^{2}$ and ${\hat{\sigma }}_{A21}^{2}$) additive genetic variances, variances of deviations of the intra- by interpopulation additive effects (σ_τ_^2^), and covariances of additive effects with their deviations (Côv_*Aτ*_) in evaluations of intra- and interpopulation half-sib progenies from cycles 0 and 3 (C0 and C3).

Traits^a^	${\hat{\sigma }}_{A11}^{2}$	${\hat{\sigma }}_{A12}^{2}$	${\hat{\sigma }}_{\tau 12}^{2}$	Côv_(*A*1*τ*12)_	Mean
	Population 1 C0				
EW	275.70 ± 159.13^b^	29.99 ± 165.24	375.11 ± 314.62	−155.205 ± 96.18	130
PH	188.74 ± 45.92	179.36 ± 49.48	229.92 ± 65.40	−59.82 ± 22.23	172.78

	Population 1 C3				
EW	393.54 ± 136.67	191.29 ± 246.82	937.45 ± 440.93	−284.92 ± 104.94	125
PH	94.91 ± 39.41	147.52 ± 47.78	−9.56 ± 68.20	15.54 ± 21.07	175.44

Traits^a^	${\hat{\sigma }}_{A22}^{2}$	${\hat{\sigma }}_{\tau 21}^{2}$	${\hat{\sigma }}_{A21}^{2}$	Côv_(*A*2*τ*21)_	Mean

	Population 2 C0				
EW	667.35 ± 137.03	660.34 ± 161.92	1237.3 ± 293.59	−311.08 ± 85.29	140
PH	−3.43 ± 32.47	197.64 ± 53.98	240.40 ± 86.49	−9.83 ± 21.73	175.31

	Population 2 C3				
EW	194.69 ± 136.99	1401.5 ± 298.35	1307.9 ± 411.11	−25.27 ± 95.15	151
PH	269.64 ± 58.11	208.71 ± 58.97	219.28 ± 102.26	−70.05 ± 32.20	178.37

^a^EW: ear weight (g/plant); PH: plant height (cm/plant).

^b^Confidence intervals for estimates.

**Table 4:** Heterosis and the direct response to reciprocal recurrent selection (RRS) in maize in the interpopulational hybrid determined on the basis of mean yield (unhusked ear weight t·ha^−1^).

Populations	Characteristics of mean values of cycle (t·ha^−1^)	
Population 1	7.19C^b^	6.92C
Population 2	7.72C	8.31B
Interpopulational hybrid 1 versus hybrid 2	8.38B	9.52A
Heterosis^a^	0.92 (12.3%)	1.9 (24.9%)

^a^Estimates of heterosis in cycles C0 and C3 were obtained from the mean yield values of the parent populations.

^b^Values with the same uppercase letters in the row are not significantly different (Scott and Knott test; *P* < 0.05).
